# WeChat official accounts’ posts on medication use of 251 community healthcare centers in Shanghai, China: content analysis and quality assessment

**DOI:** 10.3389/fmed.2023.1155428

**Published:** 2023-06-12

**Authors:** Xujian Liang, Ming Yan, Haixin Li, Zhiling Deng, Yiting Lu, Panpan Lu, Songtao Cai, Wanchao Li, Lizheng Fang, Zhijie Xu

**Affiliations:** ^1^Department of General Practice, Sir Run Run Shaw Hospital, Zhejiang University School of Medicine, Hangzhou, China; ^2^Department of Pharmacy, The First Affiliated Hospital of Xi’an Jiaotong University, Xi’an, China; ^3^The Eighth Affiliated Hospital, Sun Yat-sen University, Shenzhen, China; ^4^Department of General Practice, Tongji University School of Medicine, Shanghai, China; ^5^Department of General Practice, Taizhou Municipal Hospital, Taizhou, China; ^6^Department of General Practice, Longgang District People’s Hospital of Shenzhen, Shenzhen, China; ^7^LinCheng Health Center of Changxing County, Huzhou, China; ^8^Department of General Practice, The Second Affiliated Hospital, Zhejiang University School of Medicine, Hangzhou, China

**Keywords:** online health information, medication use, WeChat official accounts, community healthcare centers, social media

## Abstract

**Background:**

The dissemination of online health information (OHI) on medication use via WeChat Official Accounts (WOAs) is an effective way to help primary care practitioners (PCPs) address drug-related problems (DRPs) in the community. Although an increasing number of primary care institutions in China have published WOA posts on medication use, their content and quality have not yet been assessed.

**Objective:**

This study aimed to explore the general features and content of WOA posts on medication use published by community healthcare centers (CHCs) in Shanghai, China and to assess their quality of content. It also aimed to explore the factors associated with the number of post views.

**Methods:**

From June 1 to October 31, 2022, two coauthors independently screened WOA posts on medication use published throughout 2021 by the CHCs in Shanghai. Content analysis was performed to analyze their general features (format, length, and source, etc.) and content (types of medicines and diseases). The QUEST tool was used to assess the quality of the posts. We compared the differences among posts published by CHCs in central urban areas and suburban areas, and used multiple linear regression to explore the factors associated with the number of post views.

**Results:**

A total of 236 WOAs of interest published 37,147 posts in 2021, and 275 (0.74%) of them were included in the study. The median number of post views was 152. Thirty percent of the posts were reviewed by the CHCs’ staff before publication and only 6% provided information on PCPs’ consultations. The most commonly mentioned medicines and diseases in the posts were Chinese patent medicines (37.1%) and respiratory diseases (29.5%). The posts frequently provided information on indications (77%) and usage (56%) but rarely on follow-up (13%) and storage (11%). Of the posts, 94.9% had a total QUEST score < 17 (full score = 28). The median number of post views and total post quality scores did not significantly differ among the CHCs in central urban and suburban areas. In the multiple linear regression model, the number of post views was associated with scores of complementarity (B = 56.47, 95% CI 3.05, 109.89) and conflict of interest (B = −46.40, 95% CI -56.21, −36.60).

**Conclusion:**

The quantity and quality of WOA posts on medication use published by CHCs in China need improvement. The quality of posts may partially impact the dissemination effect, but intrinsic causal associations merit further exploration.

## 1. Introduction

The inappropriate use of medication is highly prevalent among patients in primary care, which could lead to drug-related problems (DRPs) that may interfere with desired health outcomes and increase treatment burden (1). The reported incidences of DRPs in primary care ranged from 8.54 to 99.16%, with the average numbers of DRPs per patient ranging from 0.58 to 7.2 (2). The cause of the resulting 6.21% of DRPs was estimated to be patient-related (e.g., poor medication adherence) (2). Most DRPs are recognized as preventable, and minimizing DRPs is essential to enhance therapeutic effects, reduce healthcare expenditures, and improve quality of life (3). To help patients avoid inappropriate use of medications and understand what to do when they have concerns about their medications, primary care practitioners (PCPs) are recommended to conduct health education on medication use in the community (4). However, PCPs in China face a great challenge in providing health education with efficiency, as there is a shortage of medical staff (5). The COVID-19 pandemic exacerbated these difficulties, as the work of prevention and control in China required extensive labor in primary care (6).

Social media has emerged as the primary source of health and medical information in recent years (7). It substantially increases the efficiency of interactions and communication between individuals and information providers. For health care professional, social media serves as an effective means to provide support and guidance to patients and to enhance collaboration and knowledge-sharing with peers (8). The population of Chinese Internet users was 1.051 billion in 2022, 99.6% of which used mobile phones to access the Internet (9). The large number of Internet users and rapid rise of social media in China offers healthcare providers new opportunities to disseminate online health information (OHI). WeChat is an instant messaging application for smartphones and the most popular social platform in China. As of June 2022, the number of monthly active users of WeChat worldwide was close to 1.3 billion, an increase of 3.8% compared to the previous year (10). The prominent market share of app users is partly attributed to its mature system of information production and dissemination. For example, individuals and organizations can register their platforms on WeChat (i.e., a specific module called WeChat Official Accounts, WOAs) to produce and disseminate information (11–14). WOAs normally focus on specific topics such as OHI and use information technology that supports multimedia messages.

Comprehensive hospitals and primary care institutions in China have increasingly registered WOAs to provide healthcare on social media. Many hospitals and community healthcare centers (CHCs) use their WOAs as the main channel to interact with patients and the public. The functions of WOAs operated by hospitals in China include hospital introduction, medical services, and visiting assistants, etc. (15). During the COVID-19 pandemic, many hospitals in China provided health information on self-protection and online counselling via WOAs (16). Our previous literature review found that many tertiary hospitals offer online pharmacy services via WOAs with the most common service being posts on medication use (17). Implementing WeChat-based pharmacy services at CHCs can help patients correctly interpret drug instructions, store medications appropriately, self-evaluate drug efficacy, and improve medication adherence (18). However, the status of primary care institutions in China that publish posts on medication use via WOAs remains unclear. The majority of studies focused on cases examples, which introduced the function of WOAs operated by their hospitals, including medication use guidance, medication instructions query, and the number of followers and post views (19). However, no study has analyzed the general features and content of WOA posts on medication use published by primary care institutions.

Meanwhile, health information on social media is unregulated, and its quality can be heterogeneous and inadequate (20). Concerns have been raised regarding the accuracy, readability, and credibility of the health information disseminated on the social media. Many articles on social media are not evidence-based and contain biased information due to the writers’ low scientific literacy and financial and intellectual conflicts of interest (21). This situation could cause misinterpretation of the OHI because most web users lack basic expertise in medicine. Low-quality health information on social media may negatively affect patient health outcomes, cause tremendous resource wastage, and hamper person-professional relationships (22). Therefore, it is essential to assess its quality, and attention should be paid to health information published through WOAs. Although the quality of information related to different health conditions on social media has been assessed in several studies (23–25), no research has been published specifically on the quality of WOA posts on medication use in China. In addition to the quality, the dissemination effect of WOA posts is also important because it relates to how much of an impact they could have (26). For example, a post with a higher number of views possibly indicates that more people are interested in the posts, which could be disseminated more efficiently (11, 27). To find strategies of disseminating health information with efficiency, the associated factors of dissemination effect (i.e., the number of views) of these posts needs to be explored.

As one of the most economically developed and densely populated regions in China, Shanghai has rich technological and medical resources, and high-quality primary care services (28). Most community healthcare centers (CHCs) operate WOAs to post online information. Analyzing the current status of primary care institutions in Shanghai publishing WOA posts on medication use could lead to an understanding of the challenges and opportunities that China faces when PCPs conduct medication education using social media. Additionally, attention has focused on the urban–rural gap in the quality of health service of primary health care. Studies have shown that the quality of primary care of CHCs in central areas is higher than that in suburban areas (29). In other studies, however, residents in suburban areas had a better experience in receiving primary care than those in central urban areas (30). The areas within the circle composed of Outer Ring Road were categorized as central urban areas by the Shanghai Municipal Government, while the remaining areas were categorized as suburban areas (31). Therefore, we hypothesized that posts published by CHCs in central urban areas would have a higher quality and greater dissemination effect. This study systematically reviewed WOA posts published by primary care institutions in Shanghai, conducted content analysis and quality assessment of the included posts, and compared the differences between the two areas. To provide decision-making support for health administrators in this field, it aimed to answer four questions regarding WOA posts on medication use:

- What general features did these posts possess and what was the content?
- What is the quality level of these posts?
- What are the differences between CHCs in central and suburban areas?
- What are the factors associated with their number of views?

## 2. Methods

### 2.1. Study sample

**Figure 1:**
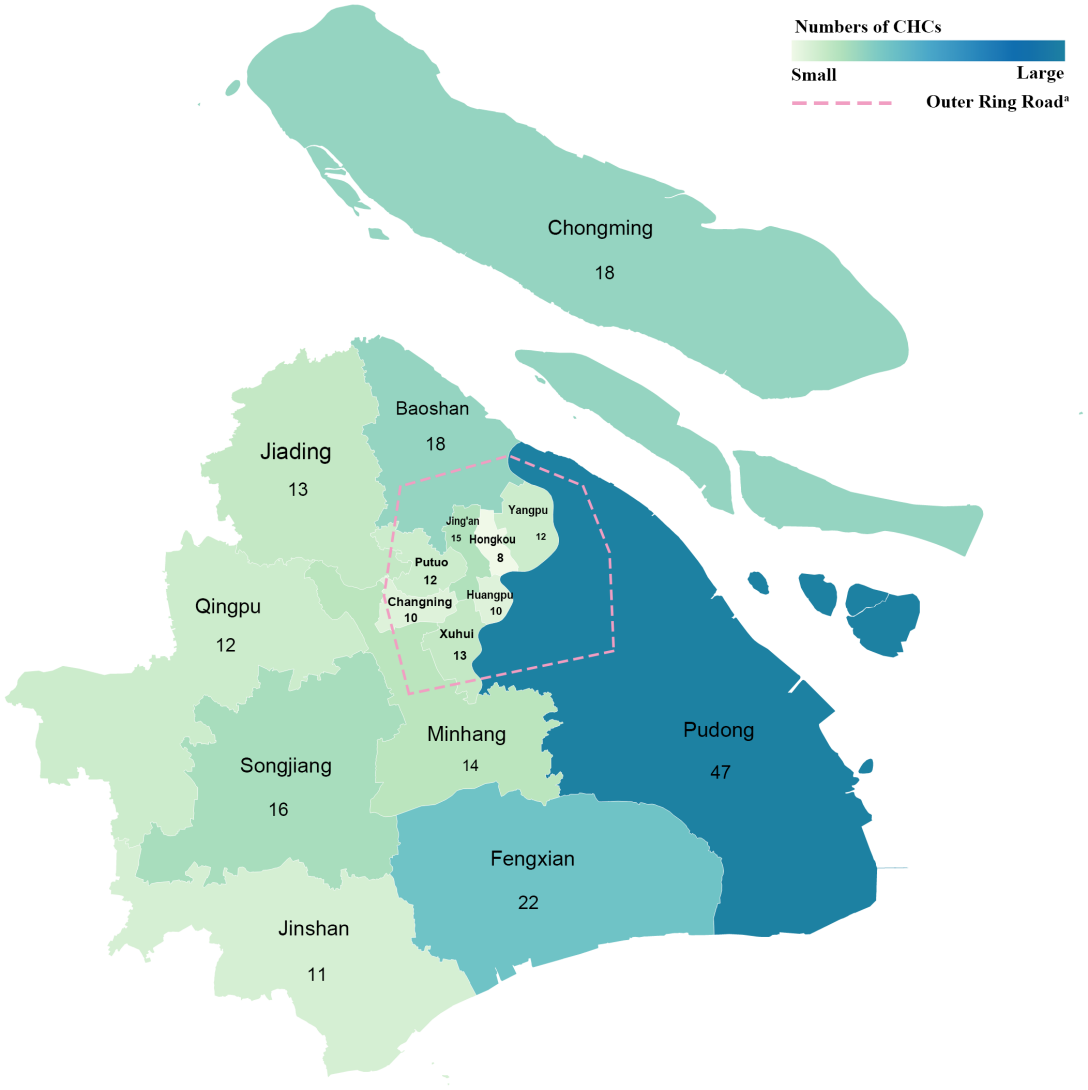
Geographic locations CHCs in Shanghai. a: according to the division made by the Shanghai Municipal Government, the central urban areas in Shanghai are located within the outer ring road, and the suburban areas are located outside of it.

The WOA posts of interest were identified using a two-step search process. First, we logged into the official website of the Shanghai Municipal Health Commission to obtain a complete list of CHCs in Shanghai (32). The locations and phone numbers of all CHCs were also obtained from the list. As of October 13, 2022, 251 CHCs have been identified in 16 districts and 107 streets in Shanghai. We located 113 CHCs in central urban areas and 138 in suburban areas using a geocode technique (Figure 1). Second, we obtained the posts by manually screening WOAs operated by CHCs in Shanghai in 2021. Posts that met the eligibility criteria were included in the content and quality assessment.

### 2.2. Eligibility criteria

The 2021 WOA posts published by the 251 CHCs in Shanghai were initially included if their content concerned the use of one or multiple medicines, and involved any one of the following elements of instructions: indications, contraindications, adverse drug reactions, usage, storage, and follow-up. We then excluded posts if (a) their content was mainly about the diagnosis and non-drug therapy of diseases, while the medication use was mentioned incidentally and lacked substantial guidance; (b) their main content was a notice or press release of activities regarding medication use (e.g., lectures on medication use to be held or already held at the CHCs); and (c) the content had been removed by the WOA publisher before screening.

### 2.3. Data collection

**Figure 2:**
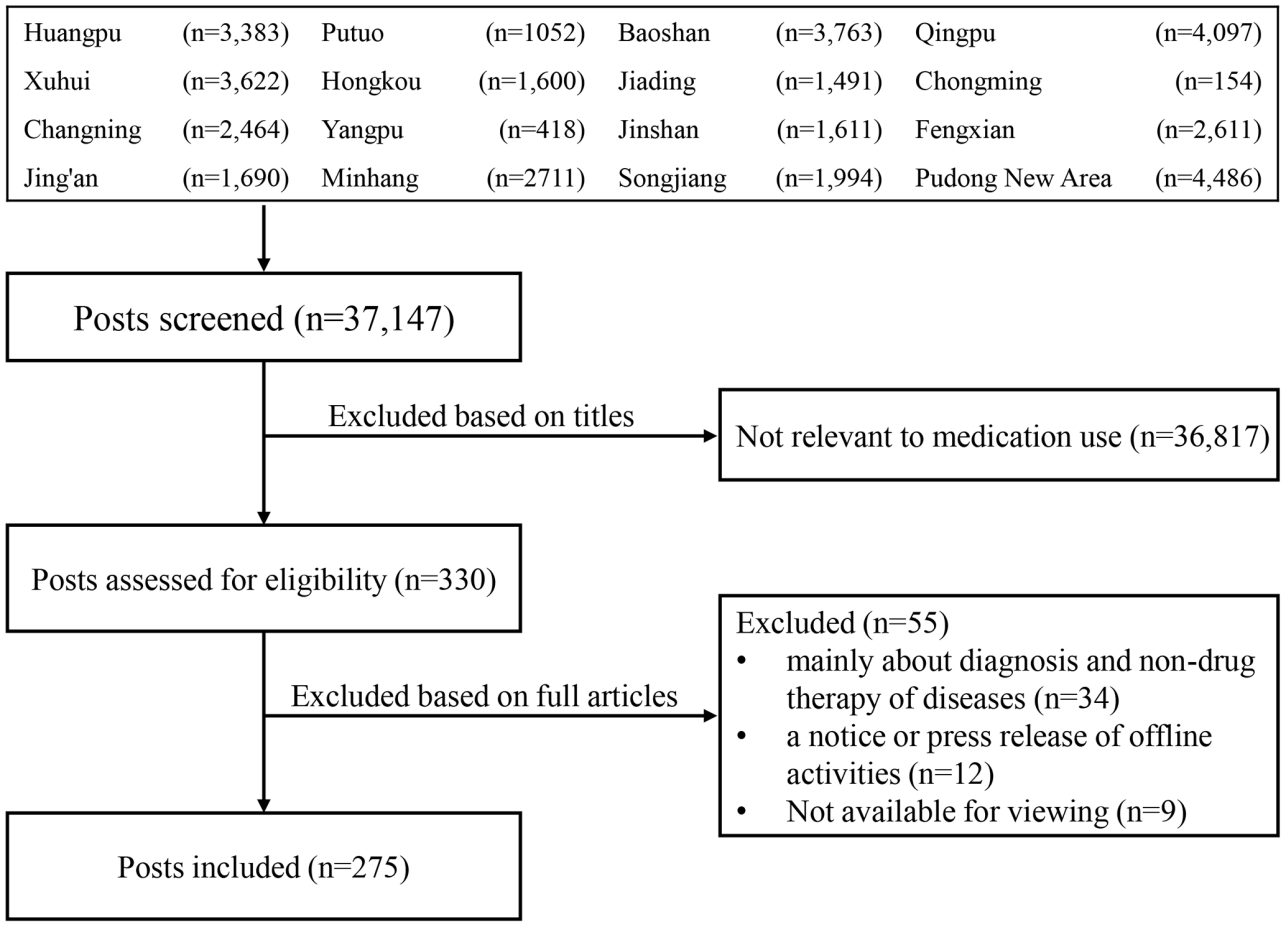
Selection procedure of the WOA posts on medication use.

WOAs were identified using the WeChat application search tool. In most cases, the names of WOAs were the same as those of their CHCs, and we could type the names of CHCs to find WOAs of interest. When searching for the names of CHCs that failed to find the targeted WOA, we contacted the CHCs to identify their WOAs by telephone. We then followed each WOA to manually screen all posts published from January 1, 2021 to December 31, 2021. All posts thus identified were independently reviewed to screen the titles by two co-authors (XL and YL) from June 1 to October 31, 2022. They subsequently screened the full texts and excluded irrelevant posts based on the eligibility criteria. They discussed and reached a consensus regarding eligibility, and a third coauthor (ZX) was consulted if the disagreement persisted. The posts were then downloaded and saved in a dedicated electronic folder. A flow diagram was used to report the search results and record the reasons for the excluded posts (Figure 2).

### 2.4. Coding procedure

We included data from any information presented in text, audio, or video formats with posts. Our research team developed and piloted a standardized data coding form. We used a Microsoft Excel spreadsheet to conduct the coding procedure (Microsoft Office Excel 2019, Microsoft Corporation, Redmond, WA, United States). The categories of codes comprised three parts (see Supplementary Table S1 for the coding rules of the content in the WOA posts and Supplementary Figure S1 for the screenshot of WOA post on medication use):

- General features: geographical location of CHCs (central urban areas/suburban areas), post source (original, reproduced, unclear), format (text, image, audio, video, or any combination of the above), whether the post provided information on PCP’s consultation (yes/no), whether the post contained references (yes/no), whether the post was reviewed by the CHCs’ staff before publication (yes/no), article length (number of Chinese characters), and number of posts.
- Content: types of medicines (e.g., antihypertensive drugs, antibiotics, etc.) and diseases (e.g., diabetes, hypertension, multiple system disease, etc.).
- Instructions for medication use: indications, contraindications, adverse drug reactions, usage, storage, and follow-up.

We initially performed a trial data coding exercise before formally coding the data. Two co-authors (XL and MY) independently coded the data from the same 30 posts, and other team members examined the codes to ensure that >90% internal consistency was achieved across all categories. Discrepancies in the coding were resolved by discussion and consensus between the two coders, and adjudicated by a third coauthor (YL) if an agreement could not be reached. Subsequently, a fourth coder (ZX) randomly selected 30% of the posts for independent coding to ensure the accuracy of the coding results.

### 2.5. Quality assessment

An OHI article’s quality is determined by its reliability, transparency, evidence-based content, and user-friendliness. To evaluate the quality of WOA posts on medication use, we utilized the Quality Evaluation Scoring Tool (QUEST), a validated tool that assesses ease of use, concision, and comprehensiveness of OHI (33). QUEST assists reviewers in quantitatively evaluating six aspects of the quality of OHI: authorship, attribution, conflict of interest, currency, complementarity, and tone. Composed of six items with weighted scores, the total QUEST score calculated by summing the scores of each item was 28.

To avoid potential evaluation bias, two coauthors (XL and HL) independently applied QUEST to rate the quality of the included posts. Similarly, the evaluation exercise was conducted before the formal assessment. Disagreements in the assessment were resolved through discussion and adjudication with a third coauthor (MY). Subsequently, a fourth coauthor (ZX) randomly selected 30% of the posts and assessed their quality independently to ensure the accuracy of the evaluation results.

### 2.6. Statistical analysis

We used quantitative descriptive analysis to report the general features and content of the included posts. The categorical variables were described as counts and/or percentages. Pearson’s chi-square test and Fisher’s exact test were used to compare the differences among CHCs in central urban and suburban areas, with an unpaired *p*-value cutoff level of 0.05 considered statistically significant. For measurement data, including article length, number of post views, and scores of each quality item, we examined their distribution using the Shapiro–Wilk test. Non-normally distributed variables were described as median and interquartile range (IQR). The differences in the CHCs in central and suburban areas were compared using the Mann–Whitney *U*-test.

Multiple linear regression models were employed to explore the linear relationship between the number of post views and potential factors (general features and quality item scores). To avoid the problem of covariance between independent variables, source and article citing references were excluded. It is because the majority of posts originally produced and citing references received high scores for authorship and attribution, respectively. In addition, currency was excluded as a constant because all posts were published in 2021 and received a full score of currency. All scores of the quality items were entered into the multiple linear regression model using the forced entry method. The models were checked for homoscedasticity, residual normality, and multicollinearity. Adjusted R Square was used to examine the fit of the model.

To demonstrate the geographic distribution of CHCs, we geocoded each CHC address using Datawrapper (Datawrapper GmbH, Berlin, Germany). We used circle size and color to indicate the number of posts published by each CHCs and the average number of post views, respectively. The Statistical Package for Social Sciences version 26.0 (SPSS Inc., Chicago, IL, United States) was used for all statistical analyses. Relevant figures were generated using GraphPad Prism version 8.3.0 (GraphPad Software, San Diego, CA, United States).

## 3. Results

Out of the 251 CHCs in Shanghai, 236 (94%) had operated their WOAs, with all CHCs in central urban areas being included. However, 15 CHCs in suburban areas had not operate WOAs as of October 31, 2022. The WOAs published 37,147 posts in 2021, 275 (0.74%) of which were included in the study. CHCs in suburban areas generated a higher percentage of posts on medicine use than those in central urban areas (1.0% vs. 0.5%, *p* < 0.001).

### 3.1. General features

**Figure 3:**
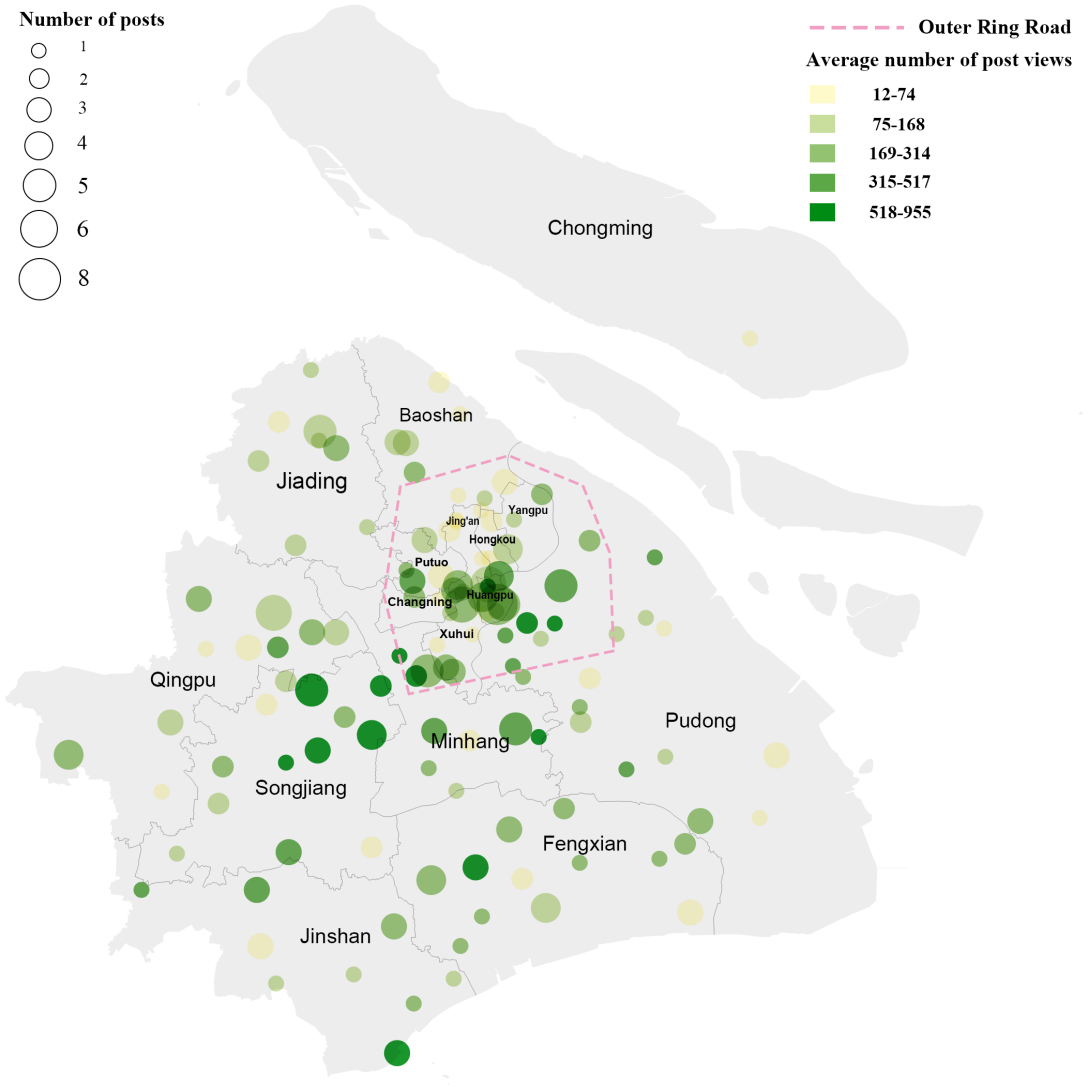
Dissemination of the posts based on CHC locations in Shanghai.

The median length of the included posts was 891.5 Chinese characters (IQR = 470), and the median number of views was 152 (IQR = 222). Nearly half of the WOA posts on medication use were originally produced by CHCs, and 20% were reproduced from other WOAs. Most posts (83%) used both images and texts to disseminate health information on medication use, yet audio (2%) and video (6%) were rarely applied. Twelve percent of posts listed references in the main text, and 30% were reviewed by the CHCs’ staff before publication. Only 6% of the posts provided information on PCPs’ consultations (Table 1). The differences between general features between WOA posts published by CHCs in suburban and central urban areas were not statistically significant, except that a higher percentage of posts were reviewed by the CHCs’ staff in CHCs in suburban areas (35% vs. 22%, *p* = 0.03). Figure 3 displays the number of posts published by each CHC and average number of views.

**Table 1:** General features of WOA posts on medication use.

General features	Total (*n* = 275)		Central urban areas (*n* = 108)		Suburban areas (*n* = 167)		*p* value
	*N*	%	*N*	%	*N*	%	
Source							0.06
Original	133	48	59	55	74	44	
Reproduced	54	20	14	13	40	24	
Unclear	88	32	35	32	53	32	
Format							0.11
Text only	25	9	9	8	16	10	
Text and Image	228	83	89	83	139	83	
Text and Image and Audio	5	2	0	0	5	3	
Text and Image and Video	17	6	10	9	7	4	
Providing information on PCPs’ consultation							0.14
Yes	18	6	10	9	8	5	
No	257	94	98	91	159	95	
Articles citing references							0.05
Yes	33	12	8	7	25	15	
No	242	88	100	93	142	85	
Articles reviewed							0.03
Yes	82	30	24	22	58	35	
No	193	70	84	78	109	65	
Article length (median, IQR)	891.5	470	915	408	876	517	0.69
Number of post views (median, IQR)	152	222	159.5	205	142	250	0.55

### 3.2. Post content on medicines and diseases

**Figure 4:**
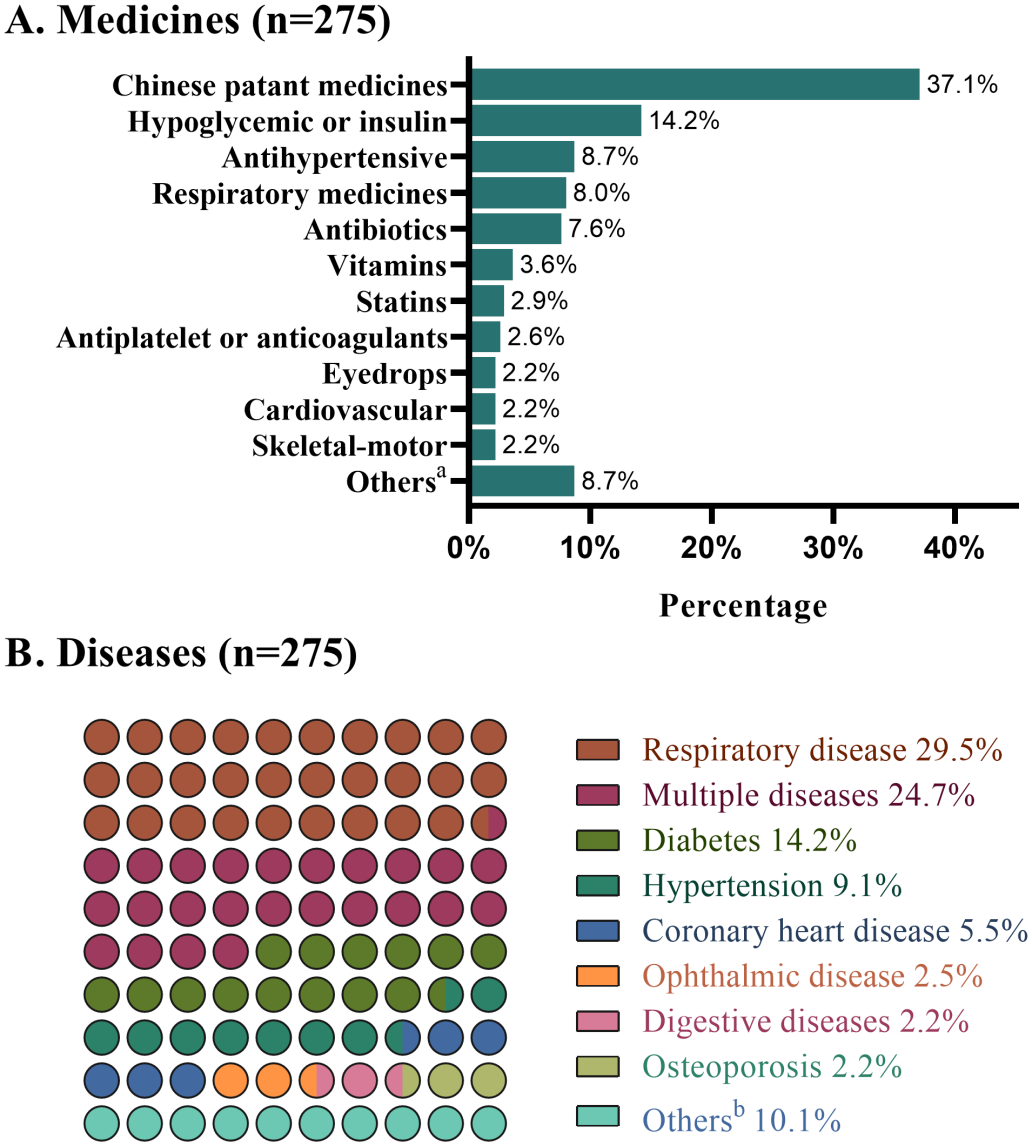
Proportions of medicines and diseases involved in the WOA posts. **(A)** Proportions of medicines. ^a^Including anti-prostate hyperplasia, levothyroxine sodium, contraceptives, thymosin, albumin, lipolytic needle, antipsychotics, and sleeping pills. **(B)** Proportions of diseases. ^b^Including prostatic hyperplasia, iron deficiency anemia, heatstroke, trauma, thyroid disease, hyperlipidemia, nutritional disease, and mental disease.

The WOA posts included in this study involved a wide variety of medications and diseases. The medicine that constituted the highest proportion was Chinese patent medicines (CPMs) (e.g., Sanfu herbal patches), which were involved in 102 (37.1%) posts. Hypoglycemia and insulin accounted for 14.2%, and each of the remaining medicines constituted less than 10% (Figure 4). As for the diseases analyzed, respiratory diseases (29.5%) were the most common, followed by posts covering multiple diseases (24.7%). Posts concerning diabetes and hypertension accounted for 14.2 and 9.1%, respectively.

### 3.3. Instructions for medication use

**Figure 5:**
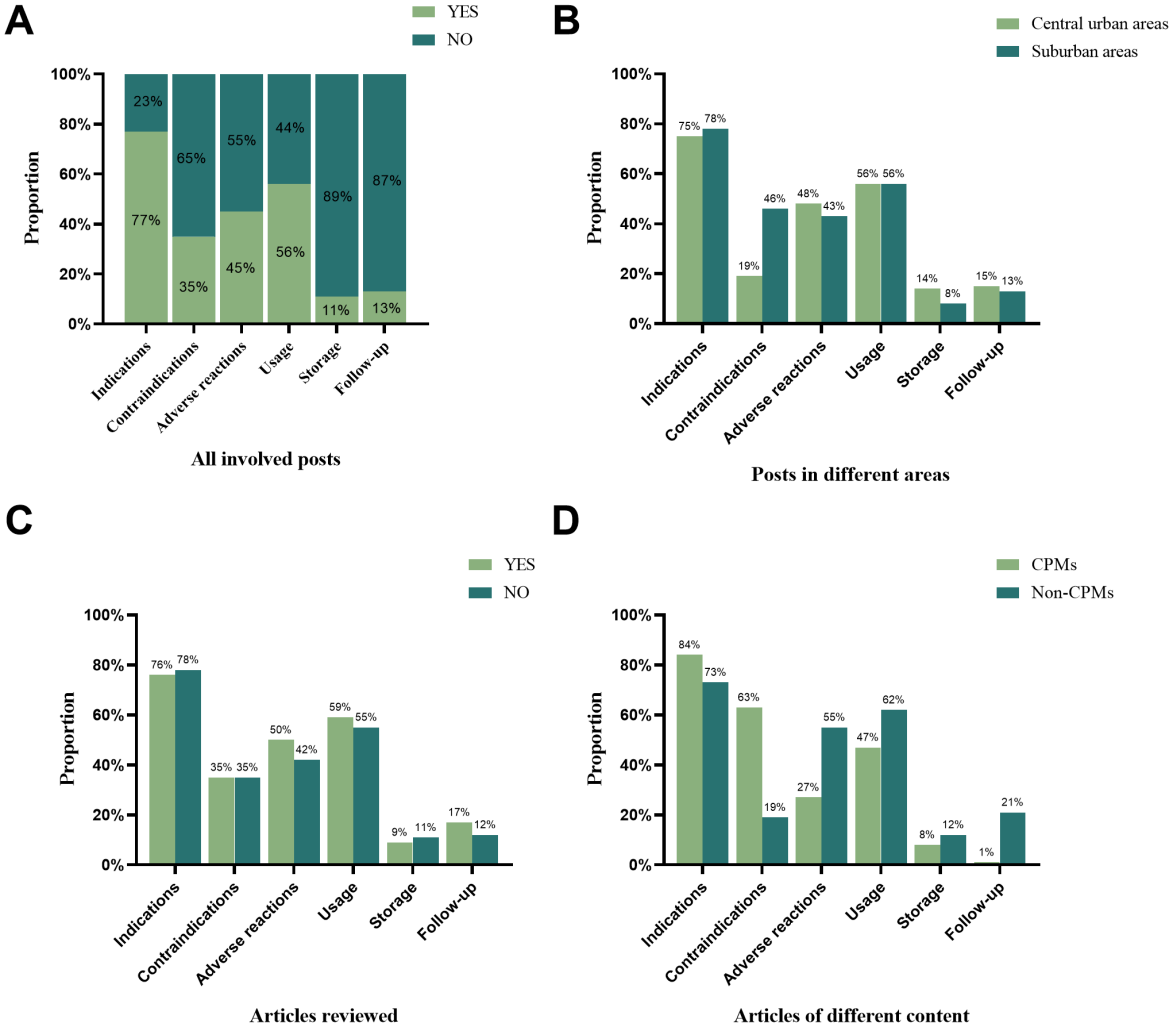
Contents of Instructions provided by WOA posts (*n* = 275). **(A)** Proportion of six different aspects of medication use instructions in all involved posts. Comparison of the proportion of central urban and suburban articles **(B)**, reviewed and non-reviewed articles **(C)**, and CPMs and non-CPMs articles **(D)** regarding medication use instructions.

As Figure 5 illustrates, posts in the study most frequently provided information on indications (77%), followed by usage (56%), but rarely on follow-up (13%) and storage (11%). Posts published by CHCs in suburban areas were more likely to introduce contraindications than those in central urban areas (46% vs. 19%, *p* < 0.001). No statistically significant differences were found between reviewed and non-reviewed articles. In addition, WOA posts on CPMs presented a higher percentage of information on indications (84% vs. 73%, *p* = 0.03) and contraindications (63% vs. 19%, *p* < 0.001) than those on non-CPMs. However, they presented fewer adverse effects (27% vs. 55%, *p* < 0.001), usage (47% vs. 62%, *p* = 0.02), and follow-up (1% vs. 21%, *p* < 0.001).

### 3.4. Quality of WOA posts

**Figure 6:**
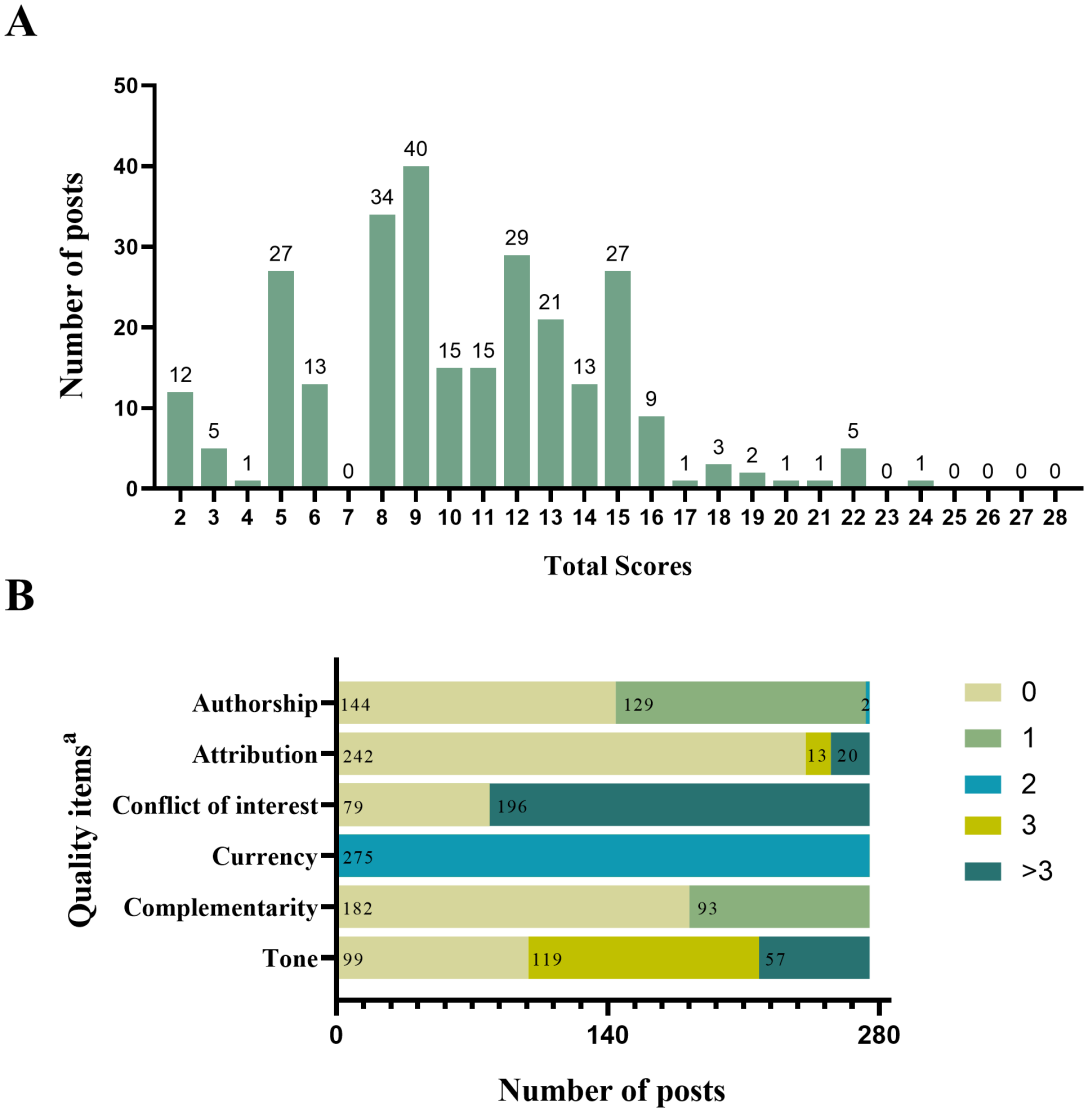
Quality scores of the WOA posts based on QUEST. **(A)** Frequency distribution of the total score. **(B)** Frequency distribution of scores of each item. ^a^Authorship and currency are rated on a scale of 0–2; attribution is rated on a scale of 0–11; conflict of interest and tone are rated on a scale of 0–6; and complementarity is rated on a scale of 0–1.

The median total score of post quality was 10 (range:2–24), and 94.9% of the WOA posts scored less than 17 (Figure 6). The median scores for authorship (0 out of 2), attribution (0 out of 11), and complementarity (0 out of 2) were particularly low. We found that 52.4% of the posts had no indication of authorship or username, and 66.2% did not provide support for the patient-practitioner relationship. Only 7.3% of the posts referenced identifiable scientific studies. The WOA posts published by CHCs in central urban areas scored higher on conflicts of interest and lower on tone than those in suburban areas, but their total scores did not significantly differ (Table 2).

**Table 2:** Scores of quality items based on QUEST (minimum to maximum achievable scores).

Scores of quality items	Total (*n* = 275)		CHCs in central urban areas (n = 108)		CHCs in suburban areas (*n* = 167)		*p* value
	Median	IQR	Median	IQR	Median	IQR	
Authorship [0–2]	0	1.0	0	1.0	1.0	1.0	0.35
Attribution [0–11]	0	0	0	0	0	0	0.051
Conflict of interest [0–6]	6.0	6.0	6.0	0	6.0	6.0	<0.001
Currency [0–2]	2.0	0	2.0	0	2.0	0	>0.99
Complementarity [0–1]	0	1.0	0	1.0	0	1.0	0.09
Tone [0–6]	3.0	3.0	3.0	3.0	3.0	3.0	0.048
Total score [0–28]	10.0	5.0	10.5	5.0	9.0	8.0	0.45

### 3.5. Factors associated with number of post views

Table 3 presents the results of the multiple linear regression on the associations between the number of post views and independent variables. Complementarity (B = 56.47, 95% CI 3.05,109.89; *p* = 0.04) was found to have a significant positive association with the number of post views, whereas there was a significant negative association for conflict of interest (B = −46.40, 95% CI −56.21, −36.60; *p* < 0.001). We did not observe any significant association between the number of post views and other variables (i.e., geographical location of CHCs, post format, articles reviewed, the presence of information on PCP’s consultation, attribution, and tone). The model was significant and explained 25.4% of the variance (*F* = 9.48, *p* < 0.001; adjusted *R*^2^ = 0.254).

**Table 3:** Associated factors of number of post views based on linear regression models.

Variables	B	SE	β	*t* value	95% CI	*p* value
**General features**						
*Geographical location of CHCs*						
Suburban areas	Ref.					
Central urban areas	−7.38	25.26	−0.02	−0.29	−57.12, 42.37	0.77
*Format*						
Text only	Ref.					
Text and image	−38.17	40.17	−0.07	−0.95	−117.26, 40.93	0.34
Text and image and audio	−73.32	98.01	−0.05	−0.75	−266.32, 119.67	0.46
Text and image and video	−12.43	60.60	−0.01	−0.21	−131.76, 106.90	0.84
**Providing information on PCPs’ consultation**						
No	Ref.					
Yes	38.24	47.96	0.04	0.80	−56.19, 132.66	0.43
**Articles reviewed**						
No	Ref.					
Yes	6.81	31.26	0.01	0.22	−54.74, 68.37	0.83
**Scores of quality items**^**a**^						
Authorship	41.28	28.15	0.10	1.47	−14.15, 96.70	0.14
Attribution	−4.88	5.89	−0.05	−0.83	−16.47, 6.71	0.41
Conflict of interest	−46.40	4.98	−0.58	−9.32	−56.21, −36.60	<0.001
Complementarity	56.47	27.13	0.12	2.08	3.05, 109.89	0.04
Tone	−9.02	5.30	−0.09	−1.70	−19.45, 1.42	0.09

a Currency was excluded from the regression analysis, as all posts had identical scores.

## 4. Discussion

### 4.1. Principal results

To our knowledge, this study is the first to systematically explore the current status of WOA posts on medication use published by CHCs in China. We identified 236 WOAs and included 275 eligible posts for analysis. We analyzed their general features and contents, assessed their quality using QUEST, and compared the differences among CHCs in central and suburban areas. Two factors were identified as associated with the number of post views. Our findings may provide an important theoretical reference in facilitating the dissemination of OHI in primary care.

### 4.2. Comparison with existing literature

Although CHCs had published numerous posts via WOAs throughout the year, it is quite alarming that the percentage of posts on medication use was less than 1%, of which only half were declared originally produced. Most WOA posts contained health information on disease management, followed by notice and news reports on CHCs. Although primary care institutions in China have been adept at using social media to disseminate OHI information, there may be a failure to recognize the significance of health education on medication use. A possible reason is the limited human resources in primary care pharmacies. In 2021, there were 167,647 pharmacists registered in primary care institutions in China, with an estimated 1.2 pharmacists per 10,000 people, which was lower than physicians (19.2/10,000) and nurses (8.1/10,000) (34). Pharmacists in China also face the constraint of their professional roles being overlooked. The main task of primary care pharmacists in China is dispensing medicines rather than patient-centered care (e.g., medication therapy management) (35). We found that only 6% of the articles provided information on PCPs’ consultation. In this context, CHCs may lack the professional knowledge and skills to produce OHI for medication use.

The WOA is a powerful tool that assists writers to use various elements when editing article content. We found that most WOA posts on medication use had both text and image formats for better readability, and a few posts used audio and video to make them easier to understand. Conversely, the reliability of WOA posts may not have received equal attention. Our results showed that the proportion of articles containing identifiable references was low. The lack of reference indicates that some content may stem from the writers’ perspectives and are not evidence-based. However, there is a conflict between readability and reliability when producing health information on social media. If it involves many professional concepts for the targeted audience, it may be difficult to read and understand regardless of its quality (36). In addition, social media users receive an abundance of information and are accustomed to fast reading, which places higher requirements on readability. Health professionals or CHC’s staff who review and modify articles before publication may help solve such problems. A total of 30% of the articles in our study reported that they had been reviewed by the CHCs’ staff before publication; however, there is still room for further improvement. In this study, we found that the most common diseases discussed in WOA posts on medication use were respiratory illnesses. This aligns with the range of illnesses observed during outpatient visits and discharges from wards of CHCs in Shanghai (37, 38). This suggests that CHCs tend to produce OHI on medication use that is relevant to the clinical needs encountered in practice. The COVID-19 pandemic has further prompted primary care practitioners to produce respiratory-related OHI on medication use (39).

Many OHI quality assessment tools, such as HONCode, DISCERN, Michigan Checklist and LIDA, have been widely applied in the past decades (40). However, these tools face challenges including extensive evaluation item sets, difficulty of use, outdatedness, and inability to provide online and quantitative results. QUEST, developed in 2018, offers a concise evaluation item set with comparable reliability and effectiveness. Its evaluation results typically align with existing tools, making it suitable for rapidly assessing a wide range of OHI, including disease prevention and treatment information. In our study, a major concern was that the overall quality of WOA posts on medication use was poor, with nearly 95% of posts receiving a total QUEST score of <17. This result was consistent with Liu et al.’s study, which found that 86.7% of WOA posts on neurological diseases were of low quality (41).

The quality assessment reveals that the reasons are multifocal. First, the health information on medication use produced by the PCPs was not comprehensive. PCPs pay a lot of attention on the indications of a variety of medicines when writing articles but tend to neglect the importance of other aspects of instructions for medication use. For example, 87% of the articles did not provide instructions for follow-up, which may have decreased their complementarity score. Second, some health information on medication use lacked supportive evidence. Our study found that CPMs held the largest proportion of WOA posts on medication use, but most have not been studied in clinical research. Thus, non-evidence-based medicine can result in low attribution scores. Third, PCPs faced difficulties in balancing their claims by caution, which was not favorable for articles that received high tone scores. Fourth, a few articles received scores of zero for authorship because a few primary care institutions routinely do not include individual names in WOA posts; therefore, the authors’ names and qualifications were omitted. In addition, PCPs’ barriers to producing high-quality WOA posts on medication use should be further explored through a qualitative approach.

Using social media is an effective approach to reach and engage with a large audience, but our findings showed that the median number of views of the included posts was relatively small compared with similar posts operated by tertiary hospitals (17). We explored the factors associated with the number of WOA post views and found that the complementarity and conflict of interest scores may have diverse associations with the number of post views. The inclusion of information on further consultation with a physician or pharmacist and endorsement of drug efficacy may contribute to the effect of post dissemination. In addition, we found that geographical location of CHC, post format, whether the post provided information on PCPs’ consultation and whether the post was reviewed were not associated with the number of post views. This result contracts with previous studies that identified article content, format, originality, and length as factors associated with user engagement (42–45). However, the findings may vary and our results should be interpreted with caution as certain potential factors were not obtained, such as the number of outpatient visits at CHCs and the number of WOA followers.

### 4.3. Implications for practice

Our findings have important implications for increasing the quantity and improving the quality of OHI for medication use in primary care settings. First, as high-quality health information on medication use in primary care in China is inadequate, we would like to call for the involvement of PCPs in producing and disseminating relevant health information. Training for knowledge and skills in this field is also needed. Establishing an effective multiteam system that involves health professionals responsible for writing/reviewing and WOA operational staff responsible for editing/dissemination could be beneficial for the sustainable progress of health education on social media. Future studies could investigate collaboration across teams in primary care institutions. Second, there is a need for primary care institutions to provide integrated health information on medication use both online and offline. As previously described, the dilemma between the reliability and readability of health information may influence patients using their medications correctly. PCPs’ counselling is a good complement to health information disseminated on social media (46). Primary care institutions could provide web-based and/or clinic-based counselling to address patient concerns about medication use promptly. This approach not only strengthens the patient-practitioner relationship but also enables better management of the COVID-19 pandemic (39). Third, given the problems of WOA posts analyzed in our study, we recommend developing practical guidelines for primary care institutions producing OHI on medication use. The content of the guidelines could incorporate general principles, ethical issues, and writing methods for OHI on medication use. More importantly, PCPs might engage a reliable checklist that assists them to screen potential problems. Thus, primary care institutions could use these guidelines as self-study materials and modify their posts based on a checklist before publication. We plan to work towards this goal with an expert panel as the next step in our research.

### 4.4. Strengths and limitations

A particular strength of this study relates to the data collection across almost all CHCs in a Chinese metropolitan city, which ensured comprehensiveness and representativeness in the analysis and interpretation of the findings. To minimize the risk of subjective bias, each step of content analysis and quality evaluation was independently conducted by at least two trained researchers and examined and arbitrated by a third researcher. All coauthors discussed and agreed upon the final results. Nevertheless, this study has several potential limitations. First, considering the developing trend of WOAs in CHCs, it focuses on posts published in 2021. The included posts may not reflect WOAs’ features and quality in the earlier stages. Meanwhile, as all posts were published within 5 years, they received a full currency score in the quality assessment using QUEST. However, our previous study showed that only 23.3% of CHCs had created WOAs in 2016, so there were a limited number of posts published at that time (47). Therefore, the features and quality of WOA posts on medication use published before 2017 might not represent the current status. Second, we included four general features and five quality item scores to explore the factors associated with the number of post views, but these may not be comprehensive and might affect the results of the associated factors. For example, the number of primary care pharmacists/physicians and annual outpatient visits of CHCs may affect the number of WOA post views, yet accurate data in this regard could be difficult to obtain during the COVID-19 pandemic. Finally, although QUEST is a validated tool with multiple strengths to assess the quality of OHI, it was not designed for OHI on medication use, which is expected to be highly professional-specific. As such tools have not yet been developed, further research can investigate the types of OHI on medication that could meet the standards for high quality.

## 5. Conclusion

The total number and proportion of WOA posts on medication use published by CHCs in Shanghai in 2021 are very limited, and their overall quality was poor. These posts involved a wide variety of medicines, the most common of which was CPMs. Most posts did not provide comprehensive instructions for medication use, particularly regarding storage and follow-up. Although CHCs in suburban areas generated a higher percentage of WOA posts on medicine use than those in central urban areas, their median number of views and total score of quality did not significantly differ. The quality of WOA posts on medication use may partially impact the number of post views, but other factors associated with the latter and their causal relationship merit further exploration.

## Data availability statement

The raw data supporting the conclusions of this article will be made available by the authors, without undue reservation.

## Ethics statement

The data used in this study were publicly available for direct collection from the WeChat application. Ethics exemption was confirmed through the institutional review board of The Second Affiliated Hospital of Zhejiang University School of Medicine. This exemption was granted because the data analyzed did not involve people or animals.

## Author contributions

XL and ZX contributed to the conceptualization, methodology and visualization. XL and MY contributed to the Software. XL, MY, and ZX contributed to the investigation. ZD and YL contributed to the data curation. XL finished the original draft preparation. LF and ZX contributed to the critical revision of the analysis and editing of the manuscript. All authors contributed to the article and approved the submitted version.

## Funding

This work was funded by the Scientific Research Fund of Zhejiang Provincial Education Department (grant: Y201941691 and Y202148336) and Zhejiang Medical and Health Science and Technology Plan Project (grant: 2023KY748).

## Conflict of interest

The authors declare that the research was conducted in the absence of any commercial or financial relationships that could be construed as a potential conflict of interest.

## Publisher’s note

All claims expressed in this article are solely those of the authors and do not necessarily represent those of their affiliated organizations, or those of the publisher, the editors and the reviewers. Any product that may be evaluated in this article, or claim that may be made by its manufacturer, is not guaranteed or endorsed by the publisher.
