# Supplementary material for: WeChat official accounts’ posts on medication use of 251 community healthcare centers in Shanghai, China: content analysis and quality assessment
Source: Front Med (Lausanne). 2023 Jun 12;10:1155428. doi: 10.3389/fmed.2023.1155428 (PMC10291264; doi:10.3389/fmed.2023.1155428)
Supplement: Supplementary file 1 [file Data_Sheet_1.docx]

Supplementary Material

# Supplementary Tables and Figures

## 1.1 Supplementary Tables

**Supplementary Table 1:** Coding rules of the content in the WOA posts

| **Codes** | **Definition** | **Coding methods** |
| --- | --- | --- |
| **General features** | | |
| Geographic location | Distinction based on the Outer Ring Road | - Central urban areas: CHCs located within the circle composed of Outer Ring Road. - Suburban areas: CHCs located outside of the circle composed of Outer Ring Road. |
| Source | Ownership of the WOA posts that indicates whether it is originally produced | - Original: The post marks the name of author and/or editor and declares the originality. - Reproduced: The post marks that it comes from other WOAs, websites, newspapers, or conference forums, etc. - Unclear: No statement of originality in the post. |
| Format | The makeup of the content in the WOA posts | - Text: The post used text to provide information for medication use. - Image: The post used images to provide information for medication use. QR codes of CHCs' WOA, and emoji are not included. - Audio: The post used audio to provide information for medication use. - Video: The post used video (e.g., video blog) to provide information for medication use. |
| Providing information on PCPs’ consultation | The WOA post describes how can a person have medical advice from the PCPs | - Yes: The post informs PCPs’ consultation schedules, clinic locations, phone numbers, or other approaches that help people consult the PCPs. - No: The post did not provide any information mentioned above. |
| Articles citing references | The article of the WOA post refers to academic materials of medication use | - Yes: The article of the post refers to as least one journal paper, clinical guideline, newspapers, conference paper or other professional material. - No: The article of the post did not list any references. |
| Articles reviewed | The post was reviewed by health professionals before publication | - Yes: The article of the post lists the name of a least one reviewer. - No: The article of the post did not list any name or information of the reviewer. |
| Article length | The total number of Chinese characters in an article of the WOA post | - The number of Chinese characters of the article is obtained by the function of word count of the WORD software. |
| Number of post views | Number of times that the post has been viewed by readers | - The data of the post is officially counted and displayed on WeChat. |
| **Content involved** | | |
| Medicines | The main medicines covered in the article | - Chinese patent medicines: San Fu Tie, San Jiu Tie, Gao Fang, etc. - Hypoglycemic or insulin - Antihypertension - Respiratory medicines: antitussive drugs, expectorants, nasal sprays, inhalants, leukotriene antagonists, antipyretic analgesics, decongestants, antihistamines - Antibiotics - Vitamins - Statins - Antiplatelet or anticoagulants - Eyedrops - Cardiovascular: nitroglycerin, digoxin - Skeletal-motor - Others: anti-prostate hyperplasia, levothyroxine sodium, contraceptives, thymosin, albumin, lipolytic needle, antipsychotics, and sleeping pills |
| Diseases | The main diseases covered in the article | - Respiratory diseases: chronic obstructive pulmonary disease, acute or chronic bronchitis, respiratory infections bronchial asthma, allergic rhinitis - Multiple diseases: disorders comprising more than one system - Diabetes - Hypertension - Coronary heart disease - Ophthalmic diseases: xerophthalmia, conjunctivitis, keratitis, refractive error - Digestive diseases: chronic gastritis, diarrhea, constipation - Osteoporosis - Others: prostatic hyperplasia, iron deficiency anemia, heatstroke, trauma, thyroid disease, hyperlipidemia, nutritional disease, and mental disease |
| **Instructions for medication use** | | |
| Indications | The conditions and/or symptoms that can be treated by the medications in the posts | - Yes: The article lists the diseases or conditions for which the drug is indicated with specific words (e.g., “benefit”, “apply to”, “suitable for use”). - No: The article does not specify the diseases. |
| Contraindications | Symptoms or conditions that make a particular treatment or medication inappropriate | - Yes: The article lists the diseases or conditions for which the drug is contraindicated with specific words (e.g., “forbidden to use”, “disable”, “prohibition”) - No: The article does not provide any information mentioned above. |
| Adverse reactions | An unintended effect of a medication that is harmful or unpleasant | - Yes: The article lists the unwanted or dangerous reactions after medication use. - No: The article does not provide any information mentioned above. |
| Usage | Information on how to use medications | - Yes: The article mentions the frequency, dosage, method, or timing of medication use. - No: The article does not provide any information mentioned above. |
| Storage | Information on how to preserve medications | - Yes: The article mentions ways to store medications properly (e.g., “avoid sunlight”, “sealed”, “moisture-proof”) - No: The article does not provide any information mentioned above. |
| follow-up | Regular consultation with a physician or pharmacist for scheduling, planning, or cycles | - Yes: The article mentions the need for further consultation with pharmacists, GPs, etc. for drug follow-up. - No: The article does not provide any information mentioned above. |

##

## 1.2 Supplementary Figures


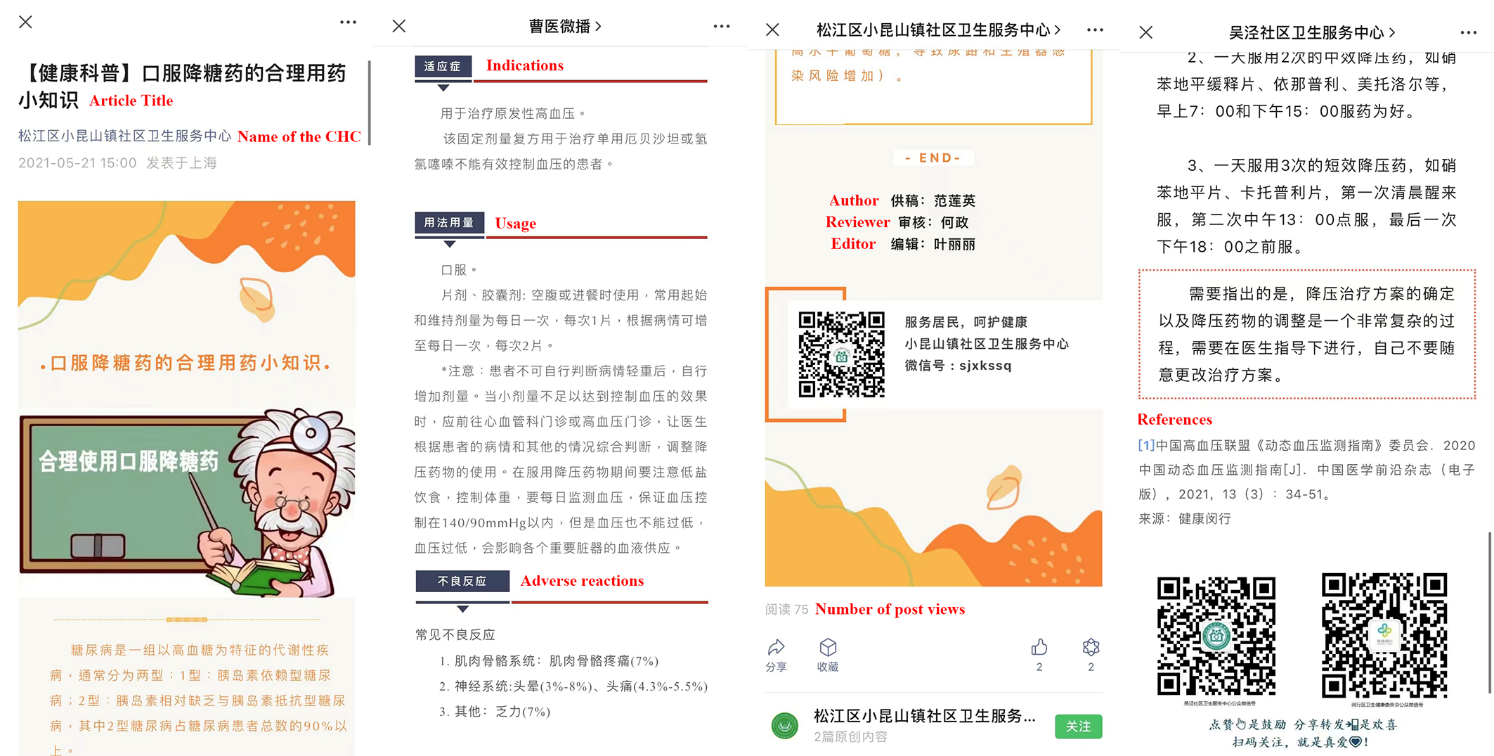


**Supplementary Figure 1.** Screenshot of WOA post on medication use.
